# Scanning Electron Microscopic Studies of Microwave Sintered Al-SiC Nanocomposites and Their Properties

**DOI:** 10.1155/2018/7546573

**Published:** 2018-01-31

**Authors:** M. A. Himyan, M. Penchal Reddy, F. Ubaid, R. A. Shakoor, A. M. A. Mohamed

**Affiliations:** ^1^Center for Advanced Materials, Qatar University, Doha, Qatar; ^2^Department of Metallurgical and Materials Engineering, Faculty of Petroleum and Mining Engineering, Suez University, Suez, Egypt

## Abstract

Al-metal matrix composites (AMMCs) reinforced with diverse volume fraction of SiC nanoparticles were synthesized using microwave sintering process. The effects of the reinforcing SiC particles on physical, microstructure, mechanical, and electrical properties were studied. The phase, microstructural, and surface analyses of the composites were systematically conducted using X-ray diffraction (XRD), scanning electron microscope (SEM), and surface profilometer techniques, respectively. The microstructural examination revealed the homogeneous distribution of SiC particles in the Al matrix. Microhardness and compressive strength of nanocomposites were found to be increasing with the increasing volume fraction of SiC particles. Electrical conductivity of the nanocomposites decreases with increasing the SiC content.

## 1. Introduction

The weight reduction in automotive industry is very critical as it improves the engine efficiency and greatly contributes to improve the fuel economy with current power trains. This is the reason that the aluminum alloys are widely used in engine and chassis components. The practice of using aluminum and magnesium castings in the automotive industry had grown rapidly in the past few years, aiding engineers to design and fabricate more fuel-efficient automobiles. Metal Matrix Composites (MMCs) are widely used in different areas of automotive, aerospace, and many other industries, along with the wide applications in the individual's daily life [1].

Among all the metal matrix composites, Al composites are the focus point in the leading up growing industries and technology. It can be used in aircraft and in cars and automotive vehicles. They also can be widely used in aerospace and military defence equipment due to their resistance and strength, which help in reducing the gas emissions and improve the future of the fuel economy, by developing a light weight material with the possible acquired performance [2–5].

AMMCs are known to possess high performance and light weight that combines all its metallic properties with ceramic properties to create light and at the same time hard materials. Many extensive researches are being done to investigate and enhance the AMMCs performance to meet the commercial conditions applications, which is one of the most important parameters that control the manufacturing process and cars leading market. The industrial demands and manufacturing conditions keep changing with time, which is the main reason to find and investigate new materials, new alloys, and new metal-metal composites [6, 7].

Most of the recent studies show an interest in metal matrix composites especially the Al composites, since they have many applications in many areas. One of many problems facing this kind of research is the type of the proper bonding between the reinforcement particles and the matrix. Researchers focus on the importance of the reinforcement bonding, chemical stability, and compatibility, in order to produce materials with proper mechanical characters that satisfy the fabrication stand points [8–12].

In general, the metal oxides (Al_2_O_3_, SiO_2_) and nitrides and carbides (B4C and SiC) were used as sintering reinforcements to improve the hardness, strength, and relative density of the metal matrix composites. Using different synthesis routes, such as powder metallurgy and spark plasma sintering, each technique has its own specific parameters and method to apply. Other manufacturing method variables that affect the final product and the AMMC composite properties include the matrix alloys, the heat and temperature treatment condition, the particle size, and volume fraction. Satisfying all the mentioned properties would result in increasing the strength of the matrix with good interfacial strength of the bonded particulates, resulting in enhanced properties [13–15].

In this study, SiC nanoparticles (0.3, 0.6, and 0.9 vol.%) reinforced Al matrix composites were synthesized using powder metallurgy process followed by microwave sintering technique. The physical, microstructure, conductivity behaviour, and mechanical properties of the composites, including their compression strength and yield strength, were investigated.

## 2. Experimental Procedure

### 2.1. Raw Materials

The commercially available elemental aluminum (APS 10*μ*m, 99.5% purity, Alfa Aesar, USA) and SiC nanopowder (45–55 nm, 99.9% purity, US Research Nanomaterials, Inc.) were used as the starting materials.

### 2.2. Synthesis of Al-SiC Nanocomposites

**Figure 1:**
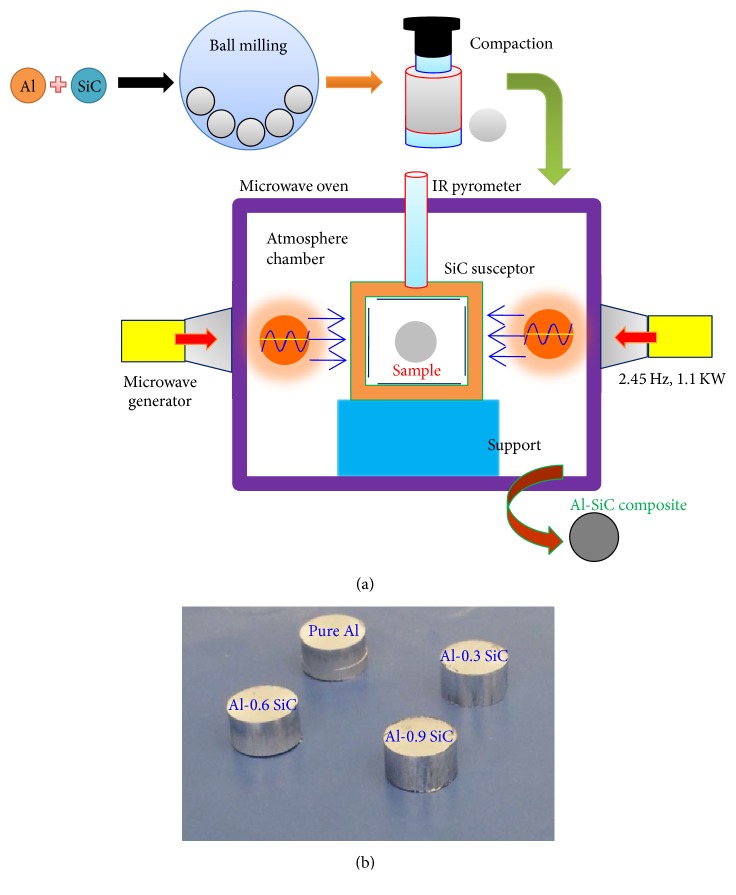
(a) Schematic diagram and (b) as-prepared samples of Al-SiC nanocomposites.

Al was used as matrix material; SiC with amount of 0.3, 0.6, and 0.9 vol.%, respectively, was used as the reinforcement in the composites. The powders were blended for 30 min using high-energy ball mill with the rotation speed of 200 rotations per minute (rpm). At this stage no balls were used to prevent particles size reduction. After blending, the mixed powders were then compacted into cylindrical pellets in hydraulic press unit by applying uniaxial compression pressure of 20 MPa and the load was maintained for two minutes under ambient conditions. The maximum pressure was optimized to get better densification and to avoid porosity in the pellets. The green compacted pellets were then subjected to sintering process. Sintering is the most important step in the synthesis of composites, as the sintering temperature and soaking time play an important role in the mechanical and physical properties of the final product. The microwave sintering process was carried out in a microwave furnace which has a silicon carbide ceramic crucible with alumina insulation as inside lining of the furnace (VB Ceramic Consultants, Chennai, India). The samples were placed in the central cavity and sintered in a microwave furnace (multimode cavity) at 2.45 GHz. SiC was selected as a microwave susceptor to aid the heating during the sintering process of the composites. The sintering process temperature was set at 550°C ± 5°C with a 30 min holding time and a heating rate of approximately 25°C/min. The sintered samples were left to slowly cool down to room temperature. The schematic diagram of the microwave sintering set-up and as-developed samples are shown in Figure 1.

### 2.3. Materials Characterization

The percentage of dimensional shrinkage was calculated by measuring the diameters of the composites before and after the sintering process. X-ray diffraction diagram of the sintered composites samples was carried out using an automated Shimadzu diffractometer. The samples were exposed to Cu K_*α*_ radiation (*λ* = 1.54056 Å) in the scanning range 30–90° at a scanning rate of 2°/m. The microstructural investigations of the sintered composites were carried out using scanning electron microscopy (SEM, Jeol Neoscope JSM 6000) equipped with energy dispersion X-ray spectroscopy (EDX). The surface roughness was measured using an Atomic force microscopy (AFM, MFP-3D Nanoindenter). Specifically, the surface roughness data are reported as root mean square (rms) of the surface. The microhardness values of the samples were measured using a Vickers's microhardness tester (MKV-h21, with applied load of 100 gf for 10 sec) and the average of at least five successive indentations for each sample was obtained. Universal testing machine-Lloyd 50 KN at a strain rate 10^−4^/sec was used to measure the compressive properties of the cylindrical pellets with a diameter of 10 mm and a height of 3 mm. The whole procedure was carried out at room temperature. Electrical conductivity analysis is a measurement method used to measure the material ability to conduct electrical current through it; a higher length will increase the resistance while higher or wider diameter will decrease the resistance, all measured by calculating the electrical resistance (*ρ*) and electrical conductivity (*σ*).

## 3. Results and Discussion

### 3.1. Sintering Shrinkage

The Al-SiC starts to contract, when a specific temperature is reached. As the temperature increases the thermal redundancy of the composites continues. The thermal redundancy stops at the sintering temperature during the dwell time, but the contracting continues as a sintering effect.

**Figure 2:**
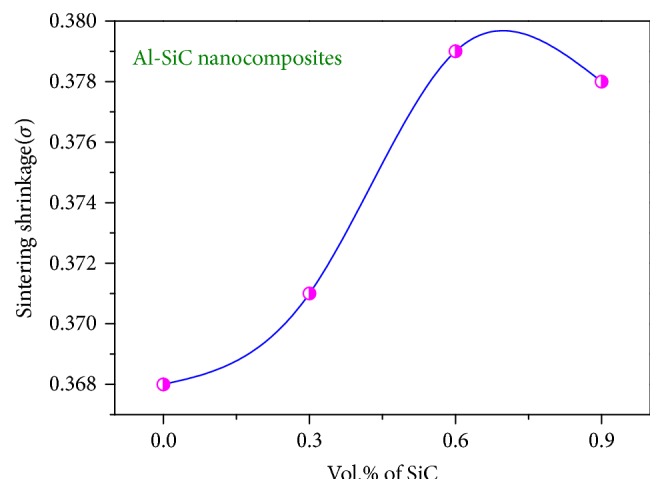
Variation of shrinkage with SiC percent.

As shown in Figure 2, Al-0.6 vol.% SiC has the highest shrinkage percentage at 550°C. However, in some cases the sintering shrinkage is different in many different directions, due to nonhomogenous microstructure of the nanocomposites or the nonspherical arrangement of composites particles [16].

### 3.2. XRD Studies

**Figure 3:**
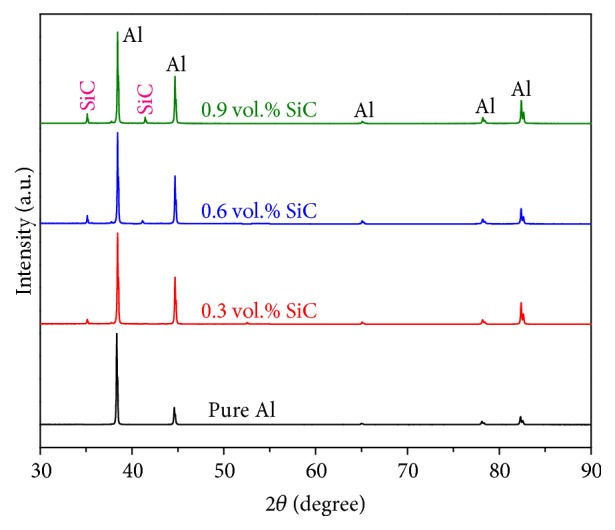
XRD patterns of Al-SiC nanocomposites.

The X-ray diffraction patterns of Al-SiC nanocomposites with different volume fractions of the reinforcement are presented in Figure 3. The individual phases of the components were identified by matching them with JCPDS data of the XRD peaks characteristic. No contamination from the vials during milling was observed and there were no diffraction peaks corresponding to the formation of any oxide. However, the microwave sintering technique used to synthesize Al-SiC nanocomposites requires relatively short time; hence it allows the retention of matrix structure of the reinforcement powder and, thereby, prevents the formation of any intermetallic phase.

The XRD results reveal that main elements present are Al (largest peak) and SiC (shorter peak).

### 3.3. SEM Analysis

**Figure 4:**
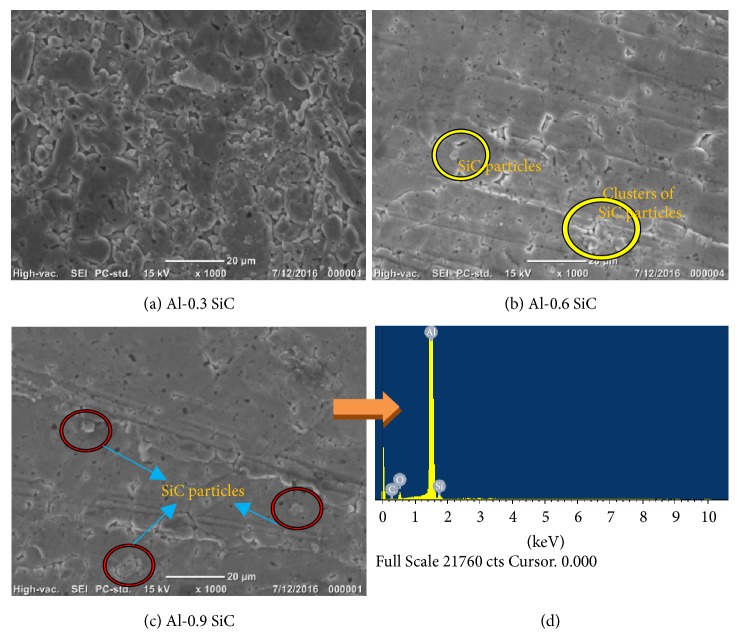
(a–c) SEM micrographs of Al-SiC nanocomposites and (d) EDX spectrum of Al-0.9 vol.% SiC nanocomposite.

The microstructure of the Al-SiC nanocomposites was investigated by SEM and the corresponding micrographs are shown in Figure 4. It can be seen that the SiC particles homogeneously distributed in the Al matrix. As shown in the SEM images the dark and light spaces correspond to Al and SiC phases, respectively.

The tendency of agglomeration at higher volume fractions of reinforcement arises, because of the large difference in the sizes of Al powder and SiC particles. The nanosized powders tend to fill in the interstitial spaces between the aluminum powders during mixing and compaction. Previous studies had reported that agglomeration of SiC particles in Al matrix resulted in the degradation of mechanical properties, as reinforcement clustering along with voids in the particles acted as preexisting cracks, limiting the stress transfer from the soft matrix to the hard reinforcements during deformation [17, 18]. However, these agglomerated sites are only observed at few locations through the matrix and a near-uniform nanoparticle distribution is noticed in the Al-SiC nanocomposite samples. This near-uniform distribution of nanoparticles promotes more even heating throughout the compacted specimens during sintering [19].

### 3.4. Roughness Analysis

**Figure 5:**
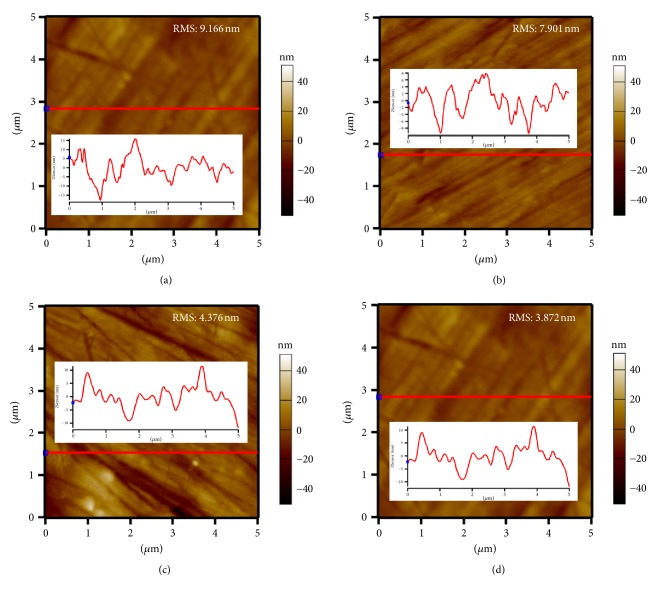
(a–d) Surface morphology of Al-SiC nanocomposites under profilometer.

The atomic force microscopic (AFM) analysis is ideal for quantitatively measuring the nanometric dimensional surface roughness and for visualizing the surface texture of the nanocomposites. Two dimensional (2D) and X-ray profile AFM images of pure Al and 0.3, 0.6, and 0.9 vol.% SiC nanoparticles reinforced Al nanocomposites are shown in Figures 5(a)–5(d), respectively. The root mean square (RMS) roughness of Al composites (9.166) gradually decreases from 7.901 nm to 4.376 nm as the reinforcement content (SiC) is increased from 0.3 to 0.9 vol.%.

### 3.5. Microhardness

**Figure 6:**
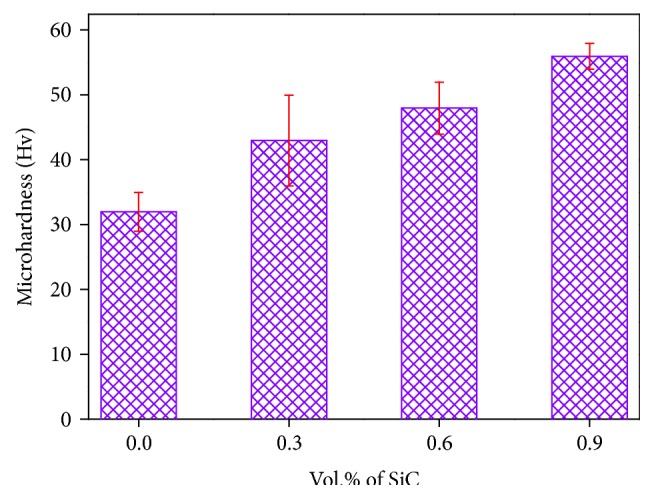
Microhardness of Al-SiC nanocomposites as a function of SiC content.

The composites hardness is a property related to the material ability to resist plastic deformation. Factors that can change or influence the disruption movement can affect Al-SiC nanocomposites hardness. The composite hardness value depends on different factors, such as the volume fraction and density of the reinforcement phase (SiC). It is evident from Figure 6 that the hardness value increased from 32 to 56 Hv with the addition of SiC particles; this result was consistent with other research [20]. This may be due to the increasing volume fraction of hard and brittle phase, resulting in the increasing of dislocation density. In addition, some interfacial reaction products have the ability to improve the wettability between SiC and Al, which in turn will promote and increase the mechanical property of the composites [20].

### 3.6. Compressive Studies

**Figure 7:**
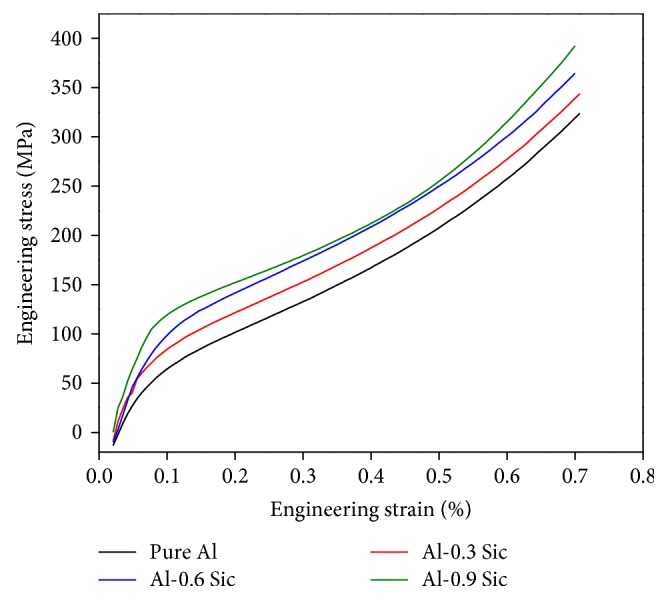
Engineering stress-strain curves for A-SiC nanocomposites under compression loading.

Figure 7 shows the engineering stress-strain curve in compression test for the microwave sintered Al-SiC nanocomposites. The composite with 0.9 vol.% of the reinforcement displays significant increases in strength with respect to the pure Al, which indicates that the SiC particles prepared by microwave sintering method have strong strengthening effect in Al matrix. The Al-0.9 vol.% SiC nanocomposite exhibited a compressive strength of 392 MPa and a yield strength of 128 MPa, respectively, which is higher than that of the Al matrix. Compressive modulus values were enhanced by the addition of SiC, and the increasing extent of the compressive modulus was 5472, 6350, and 7522 MPa achieved by various SiC addition contents (i.e., 0.3, 0.6, and 0.9 vol.%), respectively, in comparison with the pure aluminum (5243 MPa).

According to Orowan mechanism, the strengthening depends on the uniform dispersion and distribution of the reinforcing particles. Generally, nanosized particles are preferred over the micron-sized particles due to their smaller size and their effective ability in blocking the dislocation motions. Moreover, they are less prone to cracks or damage during the synthesis process of the composite [21].

### 3.7. Compressive Fractography

**Figure 8:**
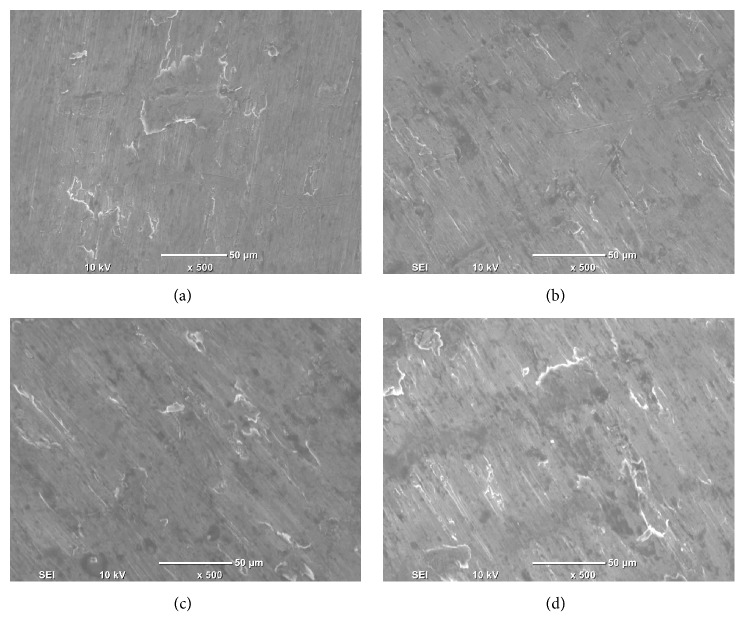
(a–d) Representative compressive fracture surface morphology of Al-SiC nanocomposites.

The compressive fractography results are shown in Figures 8(a)–8(d). In case of pure aluminum and Al-SiC nanocomposites, the fracture surface of pure Al is relatively smooth, and the shear band formation can hardly be seen in the failed samples. On the other hand, heterogeneous fracture surface along with the presence of shear bands can be found in Al-SiC nanocomposites. The plastic deformation in the nanocomposite was constrained due to the presence of dispersed second phases and comparatively large amount of grain boundaries. This led to noticeable reduction in compressive failure strain in the Al based metal matrix composites.

### 3.8. Electrical Conductivity Studies

**Figure 9:**
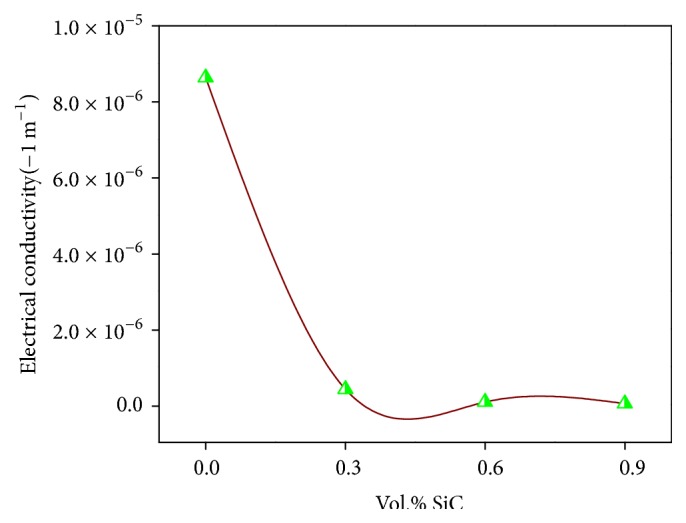
Electrical conductivity of Al-SiC nanocomposites as a function of SiC content.

In this study, the electric resistance of the Al-SiC nanocomposites was analyzed and changed into the electrical conductivity according to the ASTM standard B193-72.  Figure 9 shows a decrease in the electrical conductivity along with the increase of SiC percentage. Al-0.9 vol.% SiC composite has the lowest electrical conductivity value compared to the other composites. Al-0.3 vol.% SiC has the highest conductivity.

The electrical properties of the Al-SiC nanocomposite are vital parameters, due to their wide application in electrical circuits and supportive structures in optical devices. SiC particles hold very low electrical conductivity and because of that, their incorporation in Al alloys leads to a drastic decrease in the alloy electrical conductivity [22]. The decrease in conductivity of composite materials is basically attributed to high dislocation density near the interface and the elastic discontinuity at the interface. In addition, there is a considerable difference in thermal expansion coefficient of the matrix and the reinforcement phase. The mismatch in composites materials properties creates a high dislocation density at the interface due to the internal stresses. Such crystal defects possess a major role in scattering the electrons sites. Therefore, the electrical conductivity is easily affected by volume fraction and also by the size of reinforcement phases in composite materials [23, 24].

## 4. Conclusions

Al-SiC nanocomposites were successfully synthesized using ball milling and microwave sintering technique. The effects of SiC nanoparticles on the physical, microstructural, mechanical, and electrical properties of Al-SiC nanocomposites were investigated. The X-ray diffraction examinations did not confirm the formation of new phases; both Al and SiC peaks are shown. The microstructural examination using SEM confirms the uniform distribution of SiC nanoparticles in the matrix. With the addition of 0.9 vol.% SiC in Al matrix, an increase of 75% in hardness, 56% in yield strength, and 15% in UCS was observed compared with pure Al. Electrical conductivity values of Al-SiC composites decrease, since SiC is nonconductive material. As a result, the microwave sintered Al-SiC nanocomposites are suitable for manufacture and industrial applications.
